# Synthesis and efficacy of tetraacetyl vitamin C ester: a novel alternative to traditional vitamin C and sodium acetate in aquafeeds

**DOI:** 10.1186/s40104-026-01394-y

**Published:** 2026-04-24

**Authors:** Yanqing Li, Min Fu, Lijuan Yu, Zhen Zhang, Zhiyong Xue, Lanhui Huang, Rolf Erik Olsen, Yuanyuan Yao, Yalin Yang, Chao Ran, Chen Wang, Qianwen Ding, Zhigang Zhou

**Affiliations:** 1 China-Norway Joint Lab on Fish Gut Microbiota, Institute of Feed Research, Chinese Academy of Agricultural Sciences, Beijing, 100081 China; 2 Norway-China Joint Lab on Fish Gastrointestinal Microbiota, Institute of Biology, Norwegian University of Science and Technology, Trondheim, 7491 Norway; 3 Key Laboratory for Animal Nutrition and Feed Science of Hubei Province, Wuhan Polytechnic University, Wuhan, 430000 China; 4 Key Laboratory for Feed Biotechnology of the Ministry of Agriculture and Rural Affairs, Institute of Feed Research, Chinese Academy of Agricultural Sciences, Beijing, 100081 China; 5 Hubei Key Laboratory of Biomass Fibers and Eco-Dyeing & Finishing, College of Chemistry and Chemical Engineering, Wuhan Textile University, Wuhan, 430200 China; 6 National Key Laboratory of Agricultural Microbiology, Biotechnology Research Institute, Chinese Academy of Agricultural Sciences, Beijing, 100081 China; 7 National Collection of Livestock and Aquatic Microbes, Institute of Feed Research, Chinese Academy of Agricultural Sciences, Beijing, 100081 China

**Keywords:** Antioxidant, Intestinal health, Sodium acetate, Stability, Tetraacetyl vitamin C ester, Vitamin C-2-phosphate ester

## Abstract

**Background:**

The application of traditional vitamin C (VC) and sodium acetate (SA) in aquafeeds is hampered by significant limitations. VC suffers from poor stability during feed processing and storage, as well as low bioavailability. SA is highly hygroscopic, thermally unstable, and rapidly absorbed, leading to short duration of action. This study aimed to design, synthesize, and evaluate tetraacetyl vitamin C ester (TVCE), a novel VC-acetic acid conjugate, as a superior alternative.

**Results:**

TVCE was successfully synthesized via a one-step acetylation reaction with high yield (90%) and purity (> 95%). By optimizing the distillation recovery process, the by-product acetic acid from the reaction was efficiently recovered and recycled. TVCE demonstrated excellent lipophilicity, remarkable thermal stability (withstanding 160 °C), and was efficiently hydrolyzed by pancreatic lipase in simulated intestinal fluid, indicating its function as a prodrug. In zebrafish, TVCE enhanced systemic antioxidant capacity (increasing superoxide dismutase activity and total antioxidant capacity, while decreasing malondialdehyde content) comparably or superiorly to sodium VC-2-phosphate, even at a lower VC-equivalent dosage. In tilapia, dietary inclusion of TVCE, particularly at 0.1%, significantly improved the weight gain rate and survival rate (*P* < 0.05). Notably, despite providing only 48% of the acetate equivalent found in the physical mixture (sodium VC-2-phosphate + SA) group, TVCE still demonstrated superior performance. Furthermore, TVCE supplementation ameliorated liver and intestinal health, as evidenced by reduced serum alanine aminotransferase, diamine oxidase levels, and liver total triglyceride levels, improved tissue morphology, and an optimized gut microbiota structure characterized by an increased abundance of Bacteroidota.

**Conclusions:**

TVCE is successfully synthesized through a simple, green, and environmentally friendly process, which has successfully achieved the co-production of TVCE and acetic acid. TVCE effectively overcomes the stability and bioavailability issues of conventional VC and SA. In vivo studies demonstrated that TVCE significantly enhances growth performance, antioxidant status, and liver-intestine health in fish. These benefits are attributed to the synergistic effects of VC and acetic acid released upon hydrolysis, which results in a higher biological titer and achieving a "1 + 1 > 2" outcome. These findings establish TVCE as a promising multi-functional nutritional precursor with significant application potential in aquatic feeds.

**Supplementary Information:**

The online version contains supplementary material available at 10.1186/s40104-026-01394-y.

## Introduction

The symbiotic microorganisms in the intestines of fish, such as lactic acid bacteria, Clostridium, and Bacteroides, produce short-chain fatty acids (SCFAs) including acetic acid, propionic acid, and butyric acid by fermenting dietary fiber and other undigested carbohydrates [1]. Among them, the content of acetate is usually the highest, accounting for 60% to 85% of the total SCFAs. Acetate is not only the core product of intestinal microbial fermentation in fish, but also a key probiotic factor for maintaining the intestinal and overall health of the host [2]. Through multiple coordinated mechanisms such as energy supply, barrier protection, immune regulation, and stable flora, it builds a powerful intestinal defense system for fish, effectively enhancing their disease resistance, feed utilization rate, and growth potential, which is of great significance for the healthy and sustainable development of aquaculture [3]. However, the form of acetate products applicable to the fish feed industry has not been studied yet. Currently, it is often used in its sodium salt form, such as sodium acetate [4]. But water-soluble sodium acetate has a strong hygroscopicity, which can easily cause caking and poor fluidity during feed processing and storage, affecting the uniformity of mixing. In addition, it has poor thermal stability and is prone to weathering/decompose, resulting in the loss of active ingredients and causing inaccurate addition amounts or waste [5]. Meanwhile, it has a high polarity and is rapidly and intensively absorbed in the gastrointestinal tract of animals, resulting in large fluctuations in blood drug concentration, a short duration of action, and limited bioavailability. Therefore, there is an urgent need to develop new alternative products with higher stability and sustained-release characteristics.

Meanwhile, vitamin C (ascorbic acid, VC) is an essential water-soluble vitamin that is crucial for fish hosts and is widely used in the industry [6]. However, VC is confronted with main drawbacks in the application of aquatic animal feed, including poor stability (sensitive to light, heat and humidity, with a loss rate of 30% to 80% during processing and storage), high polarity and low absorption efficiency (bioavailability is only 20%), as well as safety risks (over-dose triggering oxidation promotion and antagonism of trace elements) [7]. This forces the industry to rely on stabilized formulations (such as VC-2-phosphate esters) and precisely regulate the addition strategy to balance efficacy and economy [8]. The applicable forms of VC in the aquatic feed industry have been fully explored in the past 30 years. Among them, VC-2-phosphate esters have become the main product form [9]. VC-2-phosphate esters are stabilized derivatives of VC, produced by esterifying the unstable enol hydroxyl groups with phosphate groups [10, 11]. Common forms include magnesium VC-2-phosphate, sodium VC-2-phosphate and VC-2-mono/polyphosphate. This chemical modification effectively enhances its thermal stability, antioxidant capacity and reduces hygroscopicity [12]. However, VC-2-phosphate esters still have strong water solubility, hygroscopicity and polarity, which greatly limits their application scenarios. Their absorption and utilization efficiency in animals is relatively low, resulting in an unsatisfactory bioavailability [13, 14]. Moreover, since the phosphorus requirement of fish hosts is much greater than the supplementation of VC-2-phosphate esters, the phosphate groups in them have no biological value.

Based on the reported dietary requirement for acetate (1.0–1.5 g/kg) in Nile tilapia (*Oreochromis niloticus*) [15] and for VC (0.05–0.4 g/kg) in species such as largemouth bass (*Micropterus salmoides*), grass carp (*Ctenopharyngodon idella*), and spotted murrel [16–18], and considering the technical bottlenecks faced by SA and VC in industrial applications, we innovatively designed a coupling product of acetate and VC. To date, no coupling products of active metabolites of the gut microbiota (such as SCFAs) and host essential vitamins (such as vitamin C) have been reported. For this purpose, the coupled product, tetraacetyl vitamin C ester (TVCE), is proposed to be obtained by using vitamin C as the framework and protecting all its easily oxidized hydroxyl groups through acetylation modification. Based on the molecular structure, we speculate TVCE features high fat solubility, good stability, low polarity, etc. This may lead to excellent biological activity, high bioavailability and safety with zero antagonism (neutral structure does not interfere with trace elements, and there is no risk of promoting oxidation when the dose is exceeded). However, the biological activity of TVCE and the interaction effect between the acetate group and the VC structural unit within its molecule are unclear. Therefore, this study intends to investigate the synthesis process and systems biological evaluation of TVCE.

## Materials and methods

### Materials

VC, zinc chloride (ZnCl_2_), sodium acetate and acetic anhydride were purchased from Sinopharm Group Chemical Reagent Co., Ltd. (Shanghai, China). Sodium VC-2-phosphate was purchased from Beijing Sunpu Biochemical and Technology Co., Ltd. (Beijing, China). Deuterated chloroform was obtained from Shanghai Macklin Biochemical Technology Co., Ltd. (Shanghai, China). Pancreatic lipase, trypsin, superoxide dismutase (SOD), total antioxidant capacity (T-AOC), malondialdehyde (MDA), diamine oxidase (DAO), and alanine aminotransferase (ALT) assay kits were purchased from Nanjing Jiancheng Biotechnology Co., Ltd. (Jiangsu, China). All other reagents and raw materials are regular ones available on the market.

The ultraviolet-visible (UV-vis) spectra of VC and TVCE were determined by the TU-1950 ultraviolet-visible spectrophotometer (Beijing Purkinje General Instrument Co., Ltd., China). Their infrared spectroscopies (FT-IR) were detected using an FT-IR spectrometer (IRTracer-100, Japan). The nuclear magnetic resonance (NMR) spectrum of TVCE was analyzed on a Bruker spectrophotometer (DPX 300, America) operating at the frequency of 300 MHz and 100 MHz. A 300 mg TVCE treated at different temperatures was mixed in a 5-mm diameter tube with 600 μL deuterated chloroform, which contained a small proportion (0.2%) of non-deuterated chloroform, and 0.03% of tetramethylsilane (TMS) generally used as a reference compound to calibrate chemical shift at 0.0 ppm (Aladdin, Shanghai, China). The NMR spectra were recorded with 32 scans. The concentration of TVCE in artificial simulation of intestinal fluid (SIF) was detected by high performance liquid chromatography (HPLC, 1220 Infinity II, Agilent, USA). The Rf values (retardation factor, specific shift value) of different compounds were obtained by thin-layer chromatography (10 mm × 20 mm) analysis with ethyl acetate and petroleum ether as developing agents.

### Synthesis of TVCE

Weigh 70.4 g of VC (0.4 mol), 612.0 g of acetic anhydride (6.0 mol), and 13.6 g of ZnCl_2_ (0.1 mol) into a 2-L flask, heat in an oil bath to 120 °C, and then stir and reflux for 5 h. After the reaction was completed, the by-product acetic acid was distilled out under reduced pressure. 500 mL water was added, and the resulting mixture was extracted with ethyl acetate (300 mL × 3), and the organic phase was combined and concentrated to obtain a white solid (124 g, content > 95%) with a yield of 90%. ^1^H NMR (CDCl_3_, 400 MHz) δ:5.50–5.47 (m, 1H), 5.39 (s, 1H), 4.43–4.38 (m, 1H), 4.33–4.29 (m, 1H), 2.28 (s, 3H), 2.26 (s, 3H), 2.07 (s, 3H), 2.05 (s, 3H); ^13^C NMR (CDCl_3_, 100 MHz) δ:170.36, 170.06, 166.16, 165.14, 164.80, 149.85, 122.05, 66.38, 62.05, 20.64, 20.52, 20.35, 20.06.

### Stability evaluation of TVCE in vitro

The digestive stability of TVCE was evaluated through artificial simulated digestion tests. TVCE (1 mg/mL) was added to the artificial SIF. Two enzyme conditions were set up: the low-dose group (trypsin 5,000 U/mL and lipase 3,000 U/mL) and the high-dose group (trypsin 5,000 U/mL and lipase 6,000 U/mL), with enzyme-free SIF as the blank control. The mixture was incubated at 28 °C (simulating fish body temperature) with shaking at 200 r/min for 4–6 h, and the reaction was then terminated in an ice bath. The content of TVCE was quantitatively analyzed by HPLC (C18 reversed-phase column, acetonitrile/water = 70:30 mobile phase, flow rate 1 mL/min, ultraviolet characteristic wavelength detection), and its digestibility was calculated. The thermal stability of TVCE was evaluated through storage tests at different temperatures. TVCE was placed in constant temperature ovens at 100, 130 and 160 °C respectively and heated for 5 min. Then, 300 mg TVCE treated at different temperatures and 600 μL deuterated chloroform were added into NMR tubes and detected. The structural changes before and after heating were compared by ^1^H NMR and ^13^C NMR to evaluate its high-temperature resistance performance, and the stacked spectra were normalized to the same vertical scale by MestReNova (Mestrelab Research, Spain), enabling direct visual comparison.

### Experimental design

**Fig. 1:**
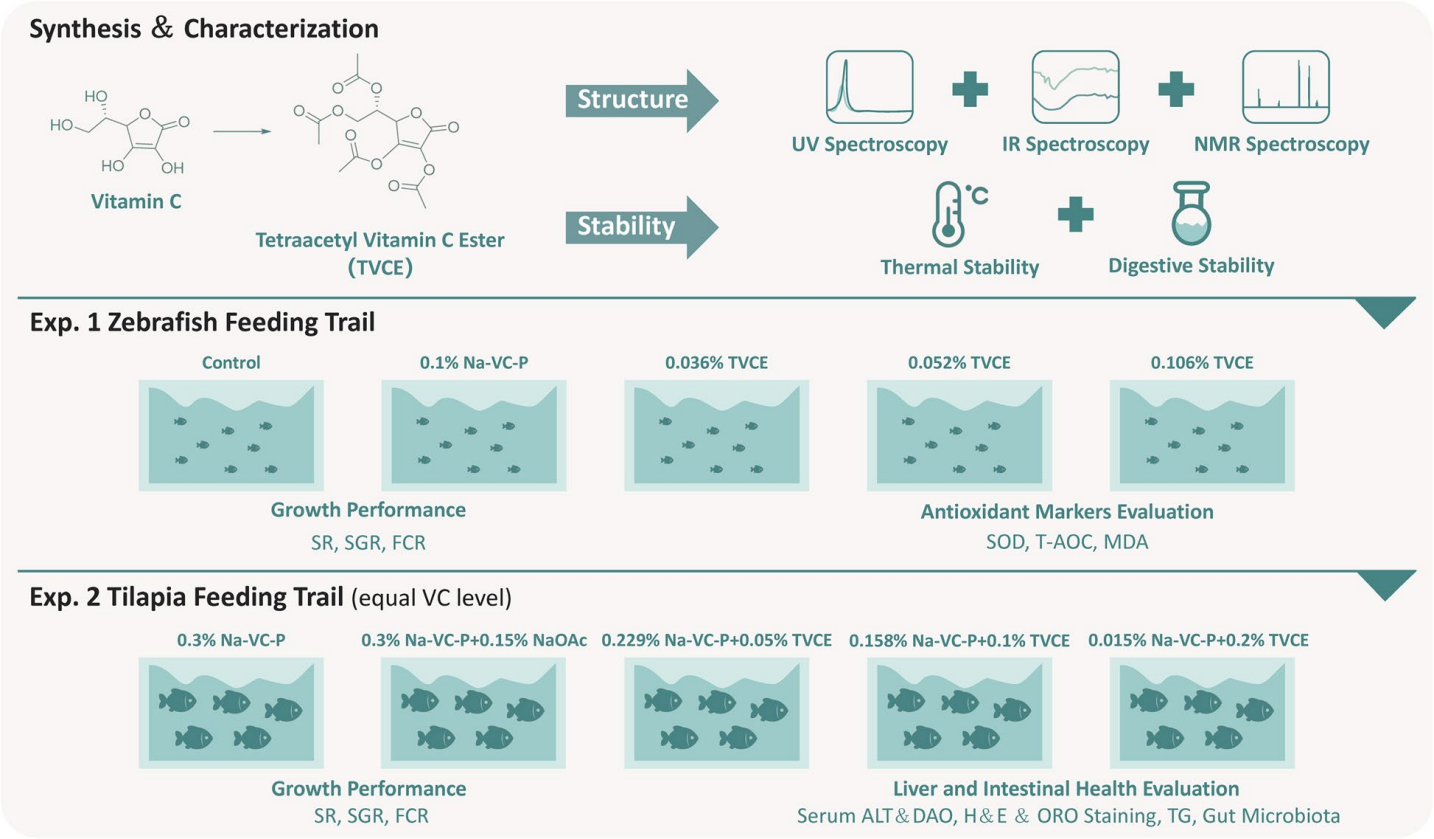
Schematic illustration of the experimental design in this study. The experimental workflow was divided into three sections: synthesis and characterization of TVCE (upper panel), assessment of its antioxidant capacity in zebrafish (middle panel), and evaluation of the effects of dietary TVCE on liver and intestinal health in tilapia (lower panel)

The experimental feed was designed based on the literature [19], with casein and gelatin as protein sources, lard and soybean oil as fat sources, and dextrin as sugar source. One-month-old zebrafish (*Danio rerio*) were supplied by the experimental base of the High-tech Industrial Park of the Chinese Academy of Agricultural Sciences (Langfang, Hebei Province). Juvenile tilapia (*Oreochromis niloticus*) with an average weight of 5.4 ± 0.01 g were sourced from tilapia breeding farms (Hainan, China). This study included a total of two aquaculture experiments (Fig. 1). In Exp. 1, five diets were prepared by adding nothing (0), 0.1% sodium VC-2-phosphate (VC0.1%), 0.036% TVCE (TVCE0.036%), 0.052% TVCE (TVCE0.052%) and 0.106% TVCE (TVCE0.106%) to the basic diet, respectively. Five experimental diets were randomly assigned to five groups of zebrafish, with four replicate tanks per diet and 10 fish per tank. All fish were reared in 7.5-L tanks for a 2‑week feeding trial. During the experiment, water temperature was maintained at 28 ± 1 °C, pH at 7.0–7.2, and a 12 h light:12 h dark photoperiod was applied. Zebrafish were hand‑fed twice daily at 08:30 and 16:30 with the corresponding diets, and feed was provided to ration. During the first week, fish were fed daily at 5% of their initial body weight, and the feeding rate was increased to 8% of the initial body weight during the second week. The substitution effect of TVCE on sodium VC-2-phosphate was evaluated by measuring the growth performance and antioxidant indicators of zebrafish. In Exp. 2, five diets were prepared by adding 0.3% sodium VC-2-phosphate (VC0.3%), 0.3% sodium VC-2-phosphate + 0.15% sodium acetate (VC0.3% + SA), 0.229% sodium VC-2-phosphate + 0.05% TVCE (TVCE0.05%), 0.158% sodium VC-2-phosphate + 0.1% TVCE (TVCE0.1%), 0.015% sodium VC-2-phosphate + 0.2% TVCE (TVCE0.2%) to the basic diet, respectively. The total VC content remained consistent among all groups. The substitution effect of TVCE on SA was evaluated by analyzing the growth performance, liver and intestinal health indicators of tilapia and the dose–effect of acetate in TVCE. In Exp. 2, each treatment included four replicate tanks with 12 tilapia per tank. Fish were reared in 90-L tanks for an 8‑week feeding trial. During the experiment, water temperature was maintained at 27 ± 1 °C, pH at 7.2–7.6, and a 12 L:12 D photoperiod was applied. The tilapia were manually fed a fixed amount of experimental diet twice daily at 08:30 and 17:30. The daily feed allowance was set at 5% of the initial body weight during the first 5 d. Thereafter, the feeding rate was increased by 2% every 5 d and maintained accordingly throughout the 8-week experimental period. The feed formulations and proximate compositions of Exp. 1 and Exp. 2 are respectively listed in Tables 1 and 2. After each feeding, the unconsumed feed was immediately collected with a plastic net, dried, and weighed.

**Table 1:** Feed nutrient composition and formula for zebrafish

Ingredient, g/kg diet	Control	VC0.1%	TVCE0.036%	TVCE0.052%	TVCE0.106%
Casein	400	400	400	400	400
Gelatin	100	100	100	100	100
Dextrin	280	280	280	280	280
Lard	30	30	30	30	30
Soybean oil	30	30	30	30	30
Lysine	3.3	3.3	3.3	3.3	3.3
VC phosphate	0	1	0	0	0
TVCE	0	0	0.36	0.52	1.06
Vitamin premix^1^	2	2	2	2	2
Mineral premix^2^	2	2	2	2	2
Monocalcium phosphate	20	20	20	20	20
Choline chloride	2	2	2	2	2
Sodium alginate	20	20	20	20	20
Zeolite powder	110.7	109.7	110.34	110.18	109.64
Total	1,000	1,000	1,000	1,000	1,000
VC content	0	0.35	0.18	0.26	0.53
Acetic acid content	0	0	0.18	0.26	0.53
Proximate composition, g/kg dry diet					
Crude protein	447	448.3	449.1	447.3	442.7
Crude fat	63.9	66.8	67.4	67.1	67.1
Ash	147.9	147.6	147.8	148.8	147.5
Moisture	109.9	111.7	112.5	111.5	111.1

^1^Vitamin premix (mg or IU/kg): 500,000 IU (international units) Vitamin A, 50,000 IU VitaminD_3_, 2,500 mg Vitamin E, 1,000 mg Vitamin K_3_, 5,000 mg Vitamin B_1_, 5,000 mg Vitamin B_2_, 5,000 mg Vitamin B_6_, 5,000 μg Vitamin B_12_, 25,000 mg Inositol, 10,000 mg Pantothenic acid, 100,000 mg Cholin, 25,000 mg Niacin, 1,000 mg Folic acid, 250 mg Biotin 10,000 mg Vitamin C

^2^Mineral premix (g/kg): 314.0 g CaCO_3_, 469.3 g KH_2_PO_4_, 147.4 g MgSO_4_·7H_2_O, 49.8 g NaCl, 10.9 g Fe (II)gluconate, 3.12 g MnSO_4_·H_2_O, 4.67 g ZnSO_4_·7H_2_O, 0.62 g CuSO_4_·5H_2_O, 0.16 g KJ, 0.08 g CoCl_2_·6H_2_O, 0.06 g NH_4_ molybdate, 0.02 g NaSeO_3_

**Table 2:** Feed nutrient composition and formula for Tilapia

Ingredient, g/kg diet	VC0.3%	VC0.3% + SA	TVCE0.05%	TVCE0.1%	TVCE0.2%
Casein	340	340	340	340	340
Gelatin	85	85	85	85	85
Dextrin	318	318	318	318	318
Fish oil	46	46	46	46	46
Soybean oil	46	46	46	46	46
Methionine	0.82	0.82	0.82	0.82	0.82
Tryptophan	1.1	1.1	1.1	1.1	1.1
Arginine	8.8	8.8	8.8	8.8	8.8
VC phosphate	3	3	2.29	1.58	0.15
Comprehensive Nutritional Premix^1^	20	20	20	20	20
Monocalcium phosphate	20	20	20	20	20
Choline chloride	2	2	2	2	2
Microcrystalline cellulose	40	40	40	40	40
Sodium acetate	0	1.5	0	0	0
TVCE	0	0	0.5	1	2
Zeolite powder	69.28	67.78	69.49	69.7	70.13
Total	1,000	1,000	1,000	1,000	1,000
Acetic acid content	0	1.05	0.25	0.5	1.0
VC content	1.05	1.05	1.05	1.05	1.05
Proximate composition, g/kg dry diet					
Crude protein	400.6	396.6	393.4	393.0	391.7
Crude fat	84.0	87.0	85.0	86.6	81.0
Ash	104.4	103.5	103.2	106.4	107.1
Moisture	82.7	79.1	79.9	80.3	78.5

^1^Comprehensive Nutritional Premix (mg or IU/kg): 500,000 IU (international units) Vitamin A, 50,000 IU VitaminD_3_, 2,500 mg Vitamin E, 1,000 mg Vitamin K_3_, 5,000 mg Vitamin B_1_, 5,000 mg Vitamin B_2_, 5,000 mg Vitamin B_6_, 5,000 μg Vitamin B_12_, 25,000 mg Inositol, 10,000 mg Pantothenic acid, 100,000 mg Cholin, 25,000 mg Niacin, 1,000 mg Folic acid, 250 mg Biotin 10,000 mg Vitamin C; 314.0 g CaCO_3_, 469.3 g KH_2_PO_4_, 147.4 g MgSO_4_·7H_2_O, 49.8 g NaCl, 10.9 g Fe (II)gluconate, 3.12 g MnSO_4_·H_2_O, 4.67 g ZnSO_4_·7H_2_O, 0.62 g CuSO_4_·5H_2_O, 0.16 g KJ, 0.08 g CoCl_2_·6H_2_O, 0.06 g NH_4_ molybdate, 0.02 g NaSeO_3_

### Sample collection

After 24 h of fasting, fish in each replicate were weighed and counted. In Exp. 1, zebrafish were anesthetized with MS-222 (tricaine methanesulfonate, 150 mg/L). Only liver and intestinal tissues were collected, and no blood sampling was performed. Collected tissues were immediately snap-frozen in liquid nitrogen and stored at −80 °C until analysis. In Exp. 2, tilapia were anesthetized using the same protocol (MS-222, 150 mg/L). Blood samples were collected from the caudal vein with a sterile syringe, kept at 4 °C, and centrifuged at 13,200 × *g* for 10 min to obtain serum. Liver, intestine, and intestinal contents were then sampled from one fish per replicate. For intestinal content collection, an additional unfasted fish per replicate was randomly selected and sampled 4 h after the last feeding.

### Growth performance analysis

Weight gain rate (WGR), specific growth rate (SGR), survival rate (SR) and feed conversion ratio (FCR) were calculate based on the literature [13].

### Biochemical parameter analysis

The serum DAO and ALT contents, and the SOD, T-AOC and MDA activities in the liver and intestine of zebrafish were determined according to the instructions of commercial assay kits (Nanjing Jiancheng Bioengineering Institute, Nanjing, China).

### Histological analysis

The liver and intestinal tissues of tilapia were successively embedded in paraffin, sectioned, stained with hematoxylin–eosin (H&E), and finally observed under a microscope (Leica DMIL-LED from Germany). Another part of liver samples were stained at room temperature with filtered Oil Red O (ORO, Sigma-Aldrich) stock solution (0.5 g of ORO in 100 mL of isopropanol) for 15 min, washed with deionized water, fixed with H&E staining, and finally images were obtained under a microscope (Leica DMIL-LED from Germany).

Liver histopathological evaluation was performed on H&E-stained sections. Hepatic steatosis, lobular inflammation, and hepatocyte ballooning were semi-quantitatively assessed using a standardized scoring system. Steatosis was graded based on the percentage of hepatocytes affected (< 5%, 5%–33%, 33%–66%, and > 66%), corresponding to scores of 0–3. Lobular inflammation was scored according to the number of inflammatory foci per 200 × microscopic field (0: none; 1: < 2 foci; 2: 2–4 foci; 3: > 4 foci). Hepatocyte ballooning was scored as 0 (none), 1 (few ballooned cells), or 2 (many cells with prominent ballooning). The overall liver pathological score was calculated as the sum of individual parameter scores.

Intestinal histopathological evaluation was performed on H&E-stained sections using a semi-quantitative scoring system. Intestinal lesions were scored based on the frequency of pathological changes observed across microscopic fields. Specifically, scores were assigned as follows: 0, no observable lesions; 1, mild lesions (1–3 affected fields per 10 microscopic fields); 2, moderate lesions (4–6 affected fields per 10 microscopic fields); and 3, severe lesions (> 7 affected fields per 10 microscopic fields).

### Hepatic triglyceride determination

Hepatic triglyceride (TG) content was determined according to a previously published method [13]. Briefly, liver tissues from two tilapia were pooled as one sample, with six replicates prepared per treatment. Lipids were extracted from liver homogenates using a chloroform–methanol (2:1) solution. After phase separation, the organic phase was collected, dried under nitrogen gas, and the extracted triacylglycerols (TAGs) were quantified using enzymatic triglyceride reagents. TG content was expressed as mg/g liver tissue.

### Gut microbiota analysis

Gut microbiota analysis was performed by Qingdao Biomarker Biotechnology Co., Ltd. (Shandong, China) using the Illumina HiSeq high-throughput sequencing platform. Briefly, total microbial DNA was extracted from intestinal contents using a commercial DNA extraction kit following the manufacturer’s instructions. The V3–V4 hypervariable regions of the bacterial 16S rRNA gene were amplified using universal primers. The purified amplicons were sequenced on the Illumina HiSeq platform. Raw sequencing data were quality-filtered, merged, and analyzed following standard pipelines described in the literature. Operational taxonomic units (OTUs) were clustered at 97% sequence similarity, and taxonomic assignment was performed based on a reference database. Alpha- and beta-diversity analyses were subsequently conducted [20].

### Statistical analysis

SPSS 22.0 (SPSS Inc., IL, USA) and origin version 2019b software (OriginLab Corporation, America) were used for data statistics and analysis. The data are expressed as mean ± SEM (standard error of the mean). One-way analysis of variance and Duncan’s multiple comparison test were used to compare the effects of different diets on fish. When *P* < 0.05, the difference is considered significant.

## Results

### Synthesis and characterization of TVCE

**Fig. 2:**
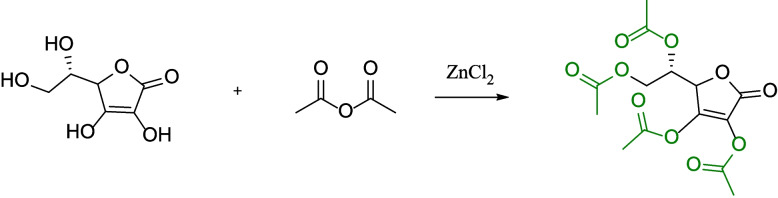
Synthetic route of TVCE

**Fig. 3:**
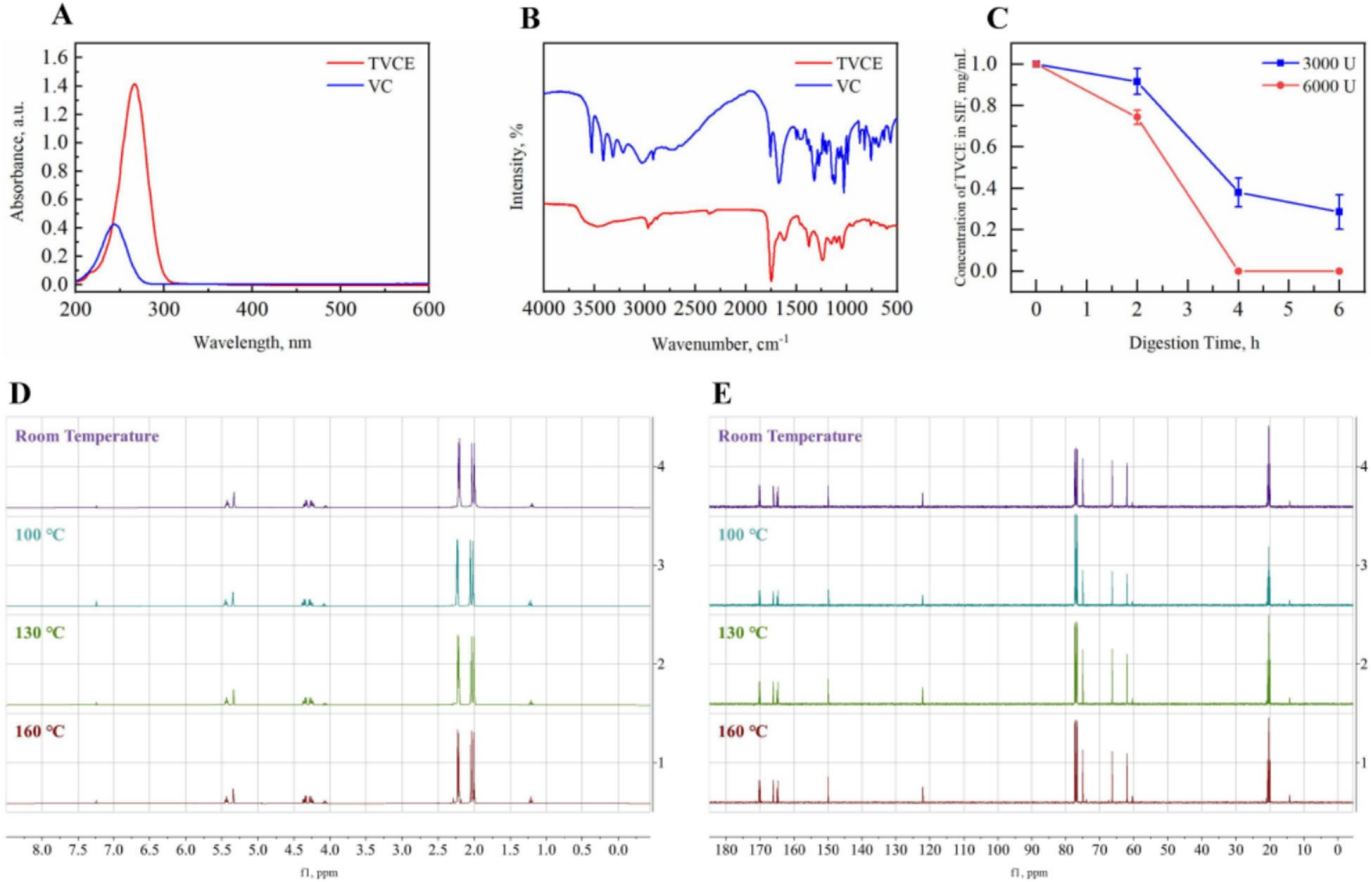
Chemical character and stability evaluation of TVCE. **A** UV-vis spectra of TVCE and VC. **B** FT-IR spectra of TVCE and VC. **C** The digestion of TVCE in SIF under 3,000 U and 6,000 U pancreatic lipase activities. ^1^H NMR (**D**) and ^13^C NMR (**E**) spectra of TVCE at room temperature (control), 100, 130 and 160 °C

TVCE was synthesized (Fig. 2) by acylating the hydroxyl groups of VC with acetic anhydride, and its yield is as high as 90%. The UV-vis and FT-IR spectra were used to analyze the chemical structural characteristics of TVCE. The maximum absorption peak of TVCE's UV-vis absorption (Fig. 3A) significantly shifts towards the red light direction (*P* < 0.05). After VC was esterified into TVCE, significant changes occurred in the infrared spectrum (Fig. 3B). The characteristic absorption at 3,600–3,200 cm^−1^ is significantly weakened or even disappears, and at the same time, strong characteristic absorption peaks at 1,750, 1,670, and 1,300–1,000 cm^−1^ appear.

### Stability assessment of TVCE

The digestive stability of TVCE was evaluated by artificially simulating intestinal digestion. In Fig. 3C, the digestibility of TVCE in SIF was positively correlated with digestion time and pancreatic lipase activities. When the pancreatic lipase activity is 6,000 U, TVCE was completely digested in just 4 h. TVCE was respectively heated in ovens at 100, 130 and 160 °C for 5 min, and its thermal stability was evaluated by the changes of ^1^H NMR and ^13^C NMR. As shown in Fig. 3D, the signals at δ 2.28 and 2.26 ppm were higher at 160 °C than at lower temperatures. More importantly, a distinct new signal appeared at δ 2.18 ppm exclusively in the 160 °C spectrum, which was absent in all other samples. In Fig. 3E, two small peaks (at δ 73.91 and 67.44 ppm) also appear in the ^13^C NMR spectrum of the TVCE sample treated at 160 °C. The NMR spectra of TVCE treated at 130 and 100 °C showed no significant difference from that of the control sample (at room temperature).

### The growth performance and antioxidant capacity of zebrafish

**Fig. 4:**
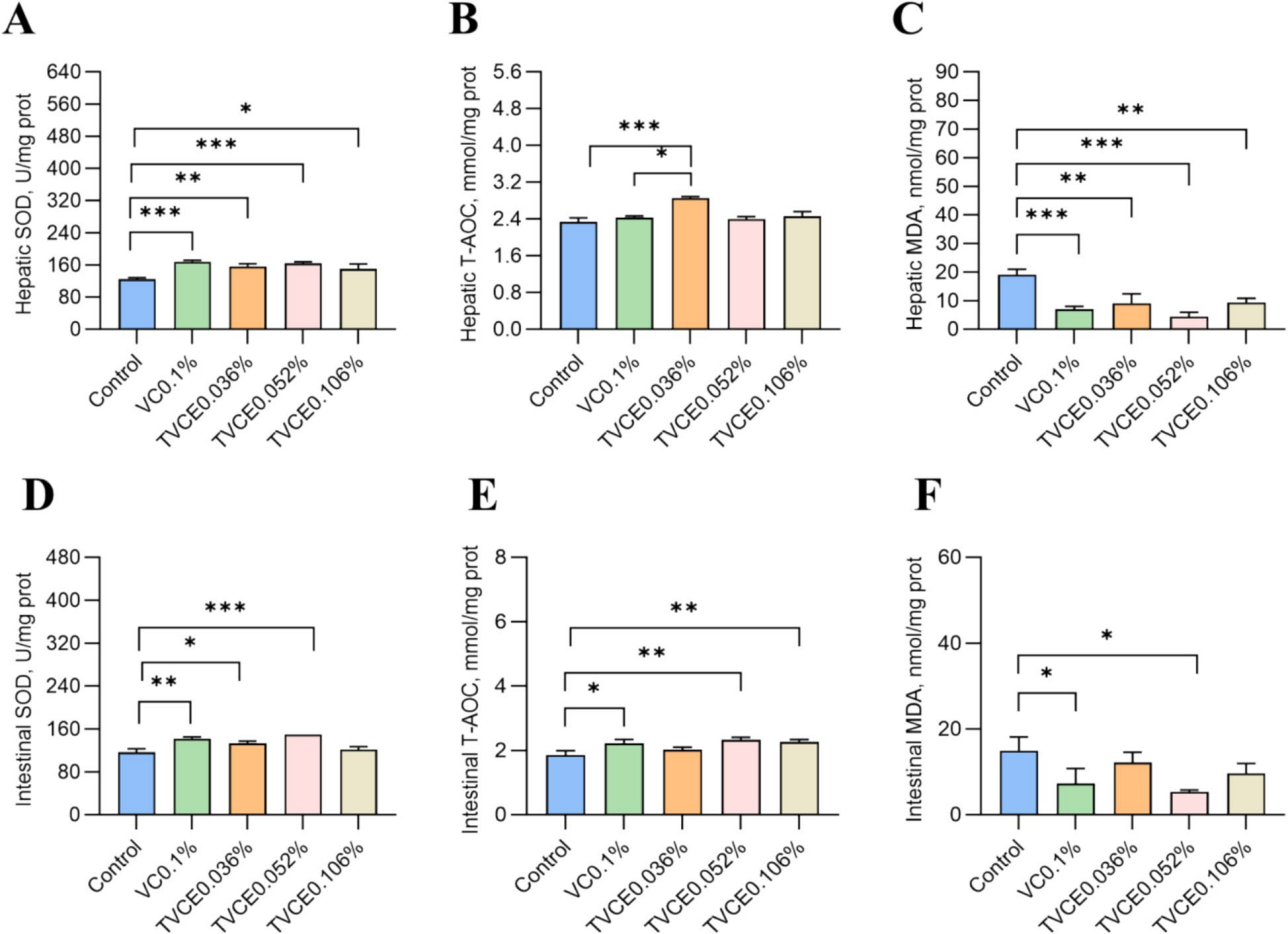
The effects of TVCE and sodium VC-2-phosphate on the SOD activities, T-AOC and MDA contents in the liver (**A**–**C**) and intestine (**D**–**F**) of zebrafish after a 2-week feeding trial. Values are presented as mean ± SEM (*n* = 4 biological replicates). Significant differences are denoted by ^*^P < 0.05, ^**^P < 0.01, and ^***^P < 0.001

As shown in Table S1, the addition of TVCE and sodium VC-2-phosphate to the diet had no significant effect on WGR, SGR and FCR of zebrafish. 0.1% sodium VC-2-phosphate and 0.036% TVCE showed a numerically lower FCR, although the differences were not statistically significant. Regarding antioxidant parameters, hepatic SOD activity was significantly higher (Fig. 4A), and MDA content was significantly lower in the VC0.1% and TVCE groups compared with the control group (*P* < 0.05, Fig. 4C). In addition, the hepatic T-AOC level in the TVCE0.036% group was significantly higher than that in the control and VC0.1% groups (*P* < 0.05, Fig. 4B). In the intestine, SOD activity (Fig. 4D) and T-AOC levels (Fig. 4E) were higher in the VC0.1% and TVCE groups compared with the control group, with the highest values observed in the TVCE0.052% group. Moreover, intestinal MDA content in the VC0.1% and TVCE0.052% groups was significantly lower than that in the control group (*P* < 0.05, Fig. 4F).

### The effects of different levels of TVCE on tilapia growth, liver and intestinal health

**Fig. 5:**
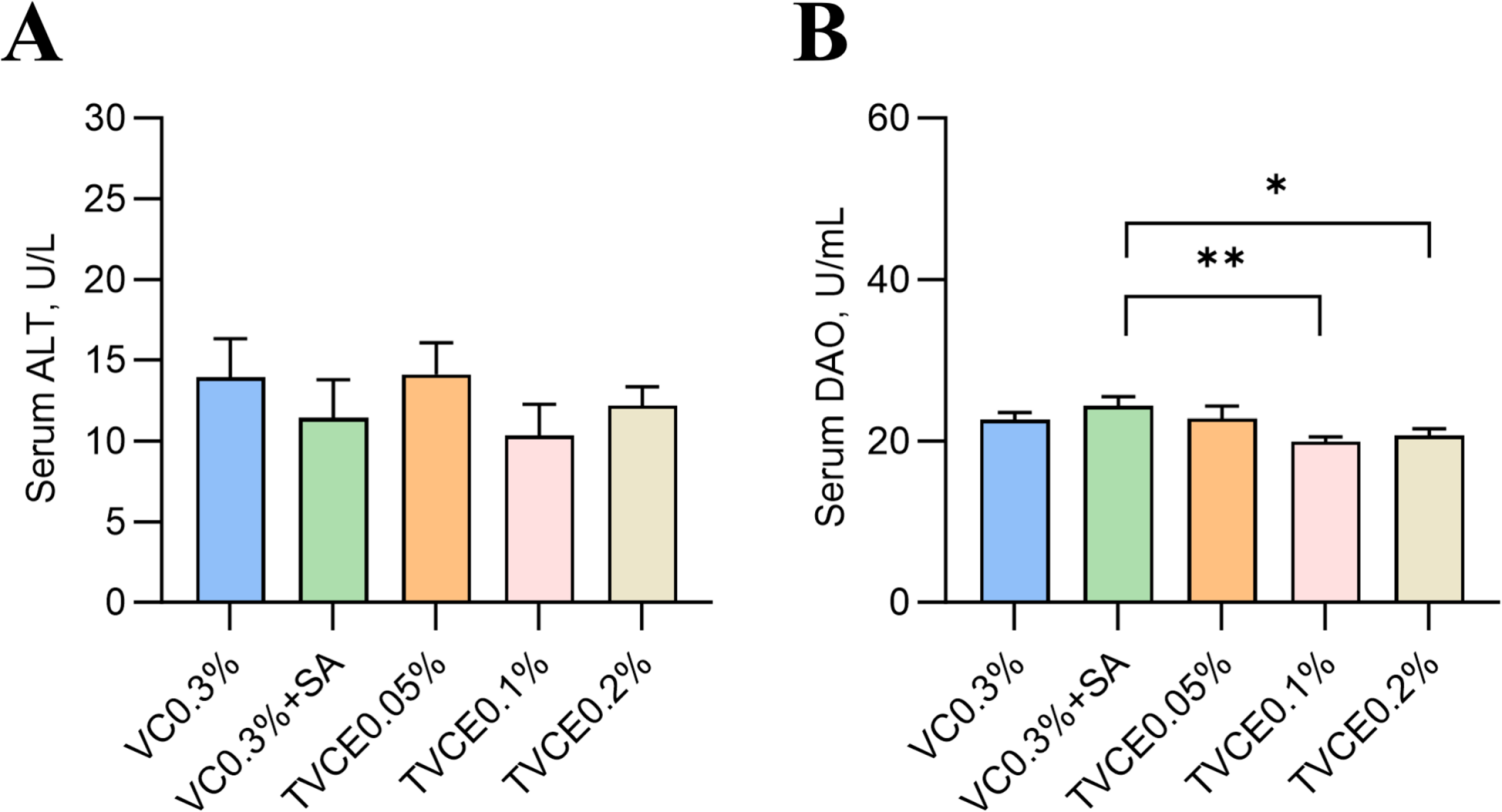
The effects of TVCE on serum ALT and DAO of tilapia fed experimental diets for 8 weeks. Values are presented as mean ± SEM (*n* = 4 biological replicates). Significant differences are denoted by ^*^P < 0.05 and ^**^P < 0.01

**Fig. 6:**
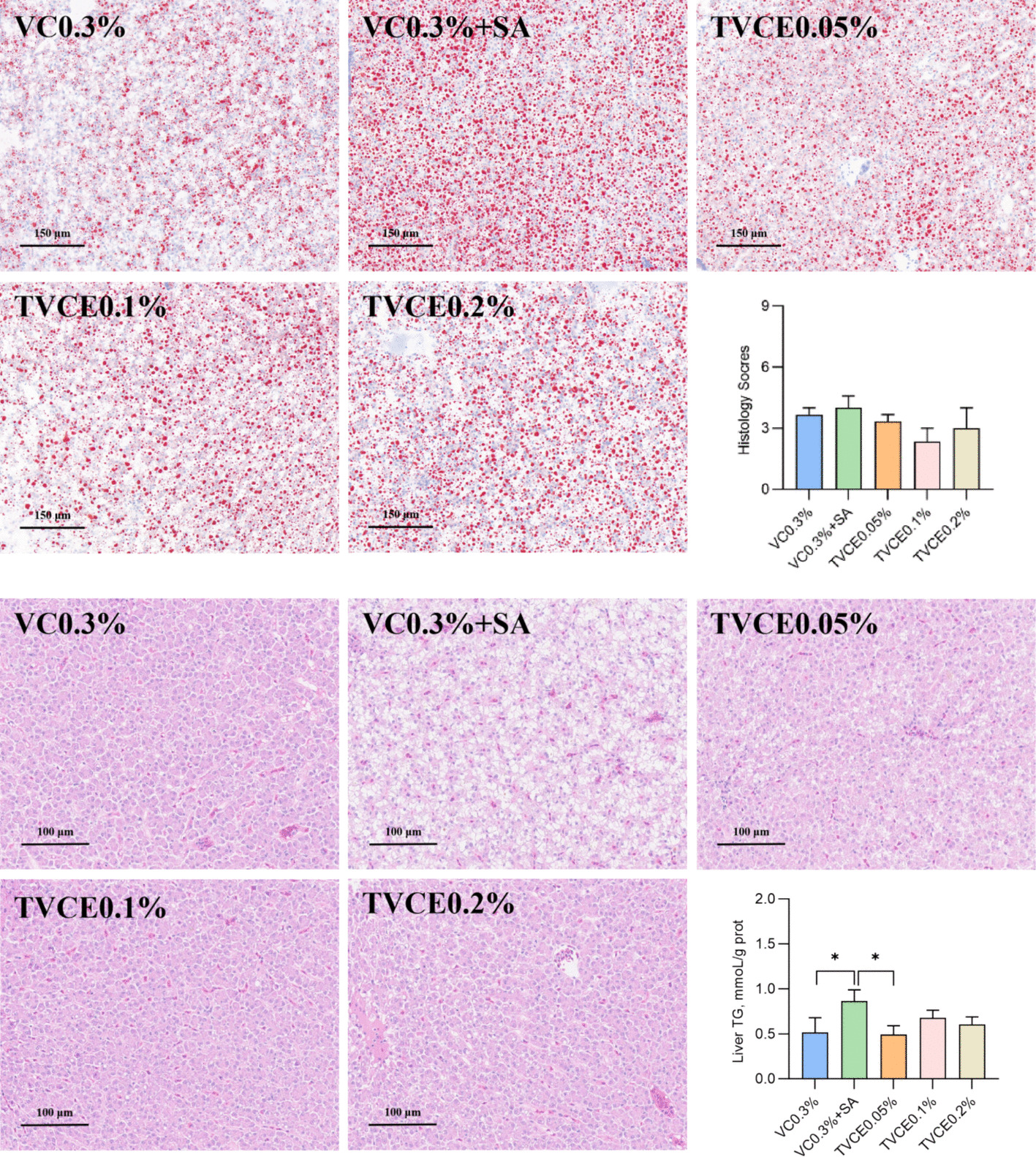
The effects of TVCE on liver morphology of tilapia fed experimental diets for 8 weeks. Liver HE sections and liver scores (top); liver oil red slices and liver TG content (bottom). Values are presented as mean ± SEM (*n* = 4 biological replicates). Significant differences are denoted by ^*^P < 0.05

**Fig. 7:**
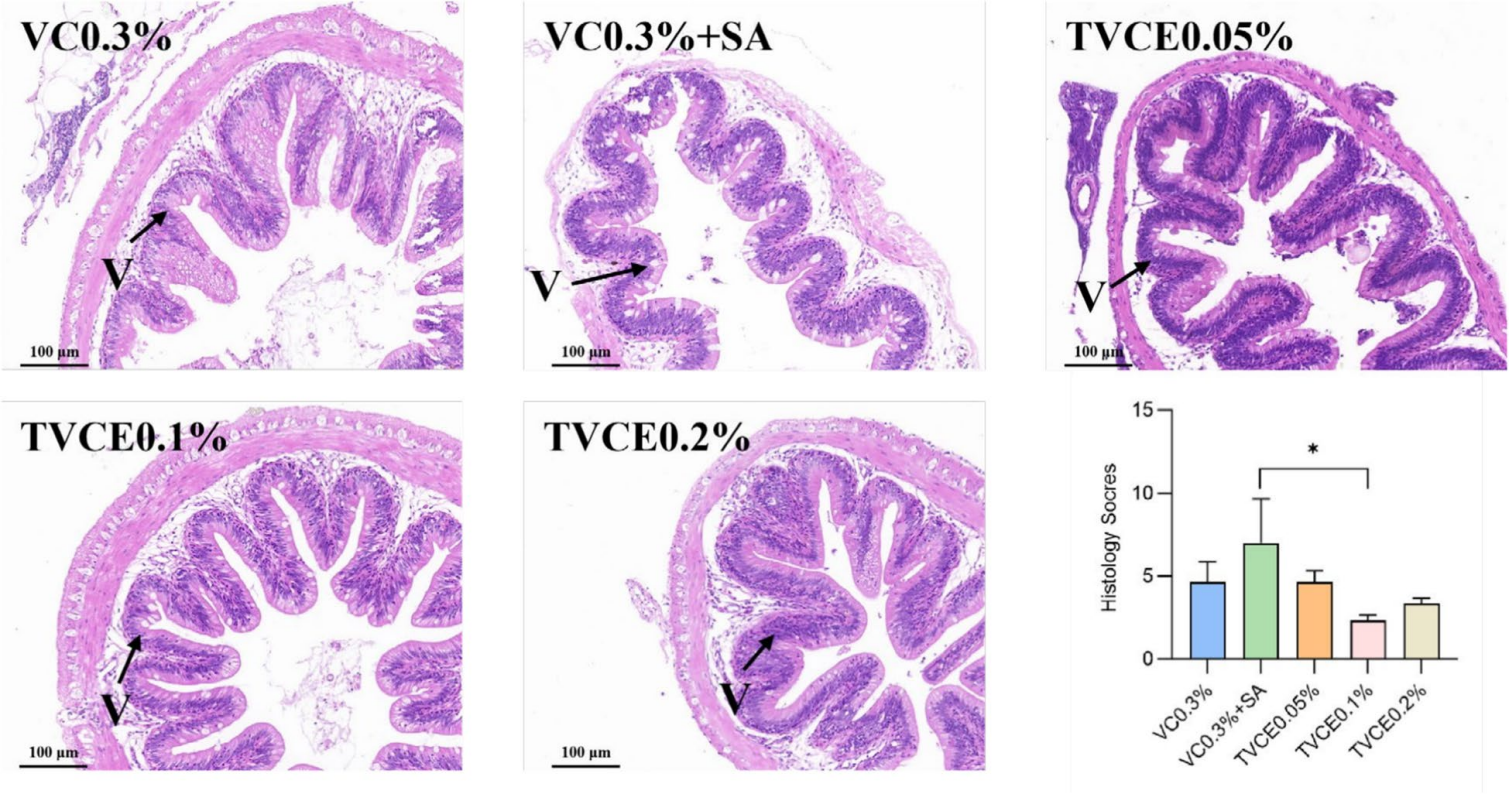
The effects of TVCE on intestinal morphology of tilapia fed experimental diets for 8 weeks. Black arrows indicate vacuolar degeneration of intestinal cells. Values are presented as mean ± SEM (*n* = 4 biological replicates). ^*^P < 0.05 indicates a significant difference

**Fig. 8:**
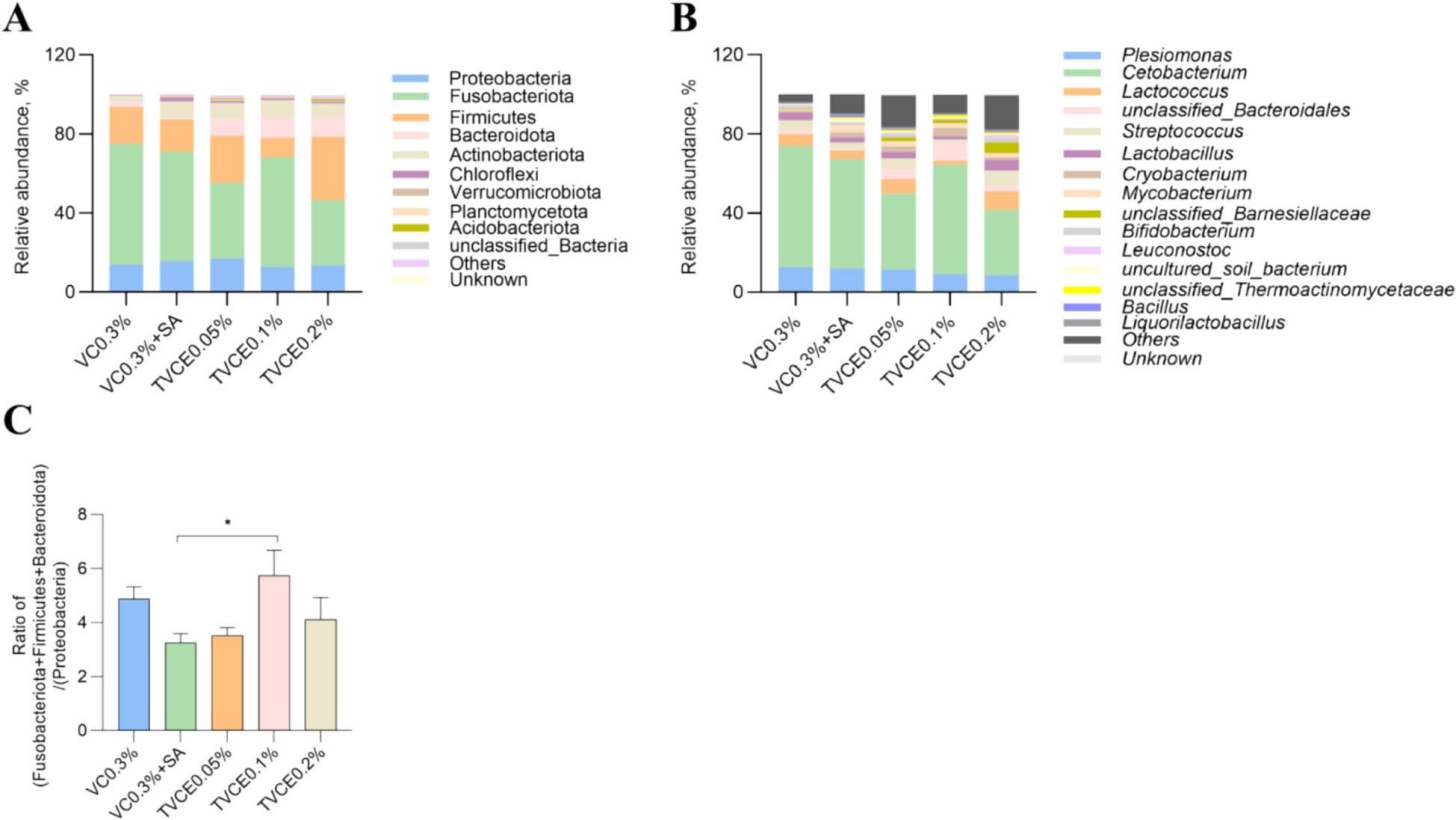
The effects of TVCE on the relative abundance of intestinal microbiota in tilapia at (**A**) phylum and (**B**) genera levels, and the ratio of the sum of Fusobacteriota, Firmicutes and Bacteroidota to Proteobacteria (**C**). Values are presented as mean ± SEM (*n* = 4 biological replicates). ^*^P < 0.05 indicates a significant difference

As shown in Table 3, there was no significant difference in FCR among all the experimental groups (*P* > 0.05). Compared with the control group, WGR was significantly higher in the VC0.3% + SA and TVCE0.1% groups (*P* < 0.05), with the TVCE0.1% group exhibiting a significantly higher WGR than the VC0.3% + SA group (*P* < 0.05). SR in the TVCE0.1% and TVCE0.2% groups was significantly higher than that in the VC0.3% + SA group (*P* < 0.05). There was no significant difference in serum ALT activities among the groups (*P* > 0.05, Fig. 5A), but these in VC0.3% + SA, TVCE0.1% and TVCE0.2% group showed a downward trend. In addition, the DAO activities in the TVCE0.1% and TVCE0.2% group were significantly lower than that in the VC0.3% + SA group (*P* < 0.05, Fig. 5B). In Fig. 6, the degree of the cell swelling and vacuolar degeneration tended to be lower in the TVCE0.1% group, whereas higher scores were observed in the VC0.3% + SA group compared with the other groups. In terms of liver pathological scores, although there was no significant difference among the treatment groups, the score of the TVCE0.1% group showed a downward trend. Oil Red O staining revealed an increased accumulation of lipid droplets in the livers of fish from the VC0.3% + SA group. Consistently, TG content in the VC0.3% + SA group was significantly higher than that in the control and TVCE0.05% groups (*P* < 0.05). H&E staining was used to evaluate the effect of TVCE on the intestinal histology of tilapia (Fig. 7). The height and the number of intestinal villi of tilapia were increased in TVCE groups, and the scores of intestinal sections in TVCE groups showed a decreasing trend. Moreover, the score in TVCE0.1% group was significantly lower than that in the VC0.3% + SA group (*P* < 0.05). As illustrated in Fig. 8, at the phylum levels, the relative abundance of Bacteroidota shows an increasing trend. There was no significant difference in the ratios of the sum of Fusobacteriota, Firmicutes and Bacteroidota to Proteobacteria among the groups the ratio (*P* > 0.05). However, compared with the VC0.3% + SA group, the ratios in the TVCE addition group showed an upward trend, and a significant difference emerged in the TVCE0.1% group (*P* < 0.05).

**Table 3:** The effects of TVCE on SR, WGR and FCR of tilapia fed experimental diets for 8 weeks

Item	**VC0.3%**	**VC0.3% + SA**	**TVCE0.05%**	**TVCE0.1%**	**TVCE0.2%**
SR, %	72.92 ± 12.5^ab^	62.5 ± 19.83^b^	79.17 ± 10.76^ab^	83.33 ± 11.79^a^	83.33^a^
WGR, %	633.60 ± 32.22^c^	723.09 ± 60.45^b^	654.60 ± 61.50^abc^	807.40 ± 74^a^	686.10 ± 39.81^abc^
FCR	1.03 ± 0.03	1.0 ± 0.03	1.06 ± 0.02	1.01 ± 0.03	1.03 ± 0.08

*SR* Survival rate, *WGR* Weight gain rate, *FCR* Feed conversion ratio

Values are presented as mean ± SEM (*n* = 4 biological replicates)

^a–c^Values with different superscript letters are significantly different (*P*< 0.05)

## Discussion

### Synthesis and property analysis of TVCE

TVCE is obtained by acylating the four hydroxyl groups of VC with acetic anhydride. Compared with VC, the main reason for the redshift in UV-vis spectrum of TVCE is that the strong electron-withdrawing induction effect of the ester group effectively enhances the degree of electron delocalization in the parent nucleus conjugated system, thereby reducing the energy required for the π → π* electron transition, and the absorption peak thus shifts towards longer wavelengths [21]. The reduction of steric hindrance and the improvement of coplanarity may also be an auxiliary factor. After VC is esterified, the characteristic absorption (3,600–3,200 cm^−1^) of the hydroxyl group (-OH) is significantly weakened or even disappears, and at the same time, strong characteristic absorption peaks (1,750, 1,670, and 1,300–1,000 cm^−1^) of the ester carbonyl group (C = O) and the carbon–oxygen single bond of the ester (C–O–C) appear. The results of NMR, UV-vis and FT-IR spectra have well confirmed the structural characteristics of TVCE.

Further, the synthesis process of TVCE has significant industrial application advantages: its core lies in the fact that it can be completed in just one reaction, with a simple and efficient process flow, and easy operation. The by-product of this reaction is economically valuable acetic acid, achieving the co-production of TVCE and acetic acid. Meanwhile, the reagents used in this process are safe and low-toxic, and the reaction medium, that is, the raw materials, are green and environmentally friendly. The generation of the three wastes is extremely small, fundamentally avoiding the residue of toxic and harmful substances. It also has excellent atomic economy and environmental friendliness. In addition, this reaction also features high yield (over 90%) and good product purity (95%). These advantages collectively endow this process with outstanding economy and scalability, laying a solid foundation for large-scale industrial production. At present, there are mainly four methods for the synthesis of VC-2-phosphate esters (Table 4): phosphorus oxychloride esterification [22], phosphate phosphorylation [23], diphosphorus pentoxide esterification, and VC phosphorylase catalytic [12] method. The phosphoric acid esterification method of phosphorus oxychloride has disadvantages such as complex process and many by-products. This method requires a large amount of pyridine as a solvent and involves protection/deprotection operations as well as cumbersome purification steps. In addition, during the reaction process, by-products such as L-ascorbic acid-3-phosphate and L-ascorbic acid-2-pyrophosphate are prone to be generated, which affect the yield and purity of the target product [22]. The phosphate phosphorylation method has the advantages of low pollution and simple operation, but there are still obvious technical bottlenecks, including low effective content of the product, poor curing stability, dark color and poor uniformity of the finished product, etc. Further process optimization is urgently needed [23]. The phosphoric acid esterification method of diphosphorus pentoxide is similar to that of phosphorus oxychloride. Although the enzymatic catalytic method has mild and green reaction conditions, it is difficult to induce in the early stage and takes a long time. Moreover, it is still in the experimental stage and has not yet been put into large-scale production [12]. Therefore, these synthetic methods are characterized by complex operation, highly toxic reagents used, numerous by-products, high purification difficulty, environmental pollution, high cost or low product quality, making them difficult to achieve large-scale production. It is evident that the acetylation synthesis method of TVCE is far superior to those of VC-2-phosphate esters. More importantly, synthesis processes and characteristics of VC-2-phosphate esters and TVCE are very different. Tables 5 and 6 present the synthesis processes and characteristic differences between VC phosphate esters (taking sodium VC-2-phosphate as an example) and TVCE. Table 4 conducts a multi-dimensional comparison of the synthesis processes between sodium VC-2-phosphate and TVCE. In terms of reaction steps, sodium VC-2-phosphate requires 2–4 steps, while TVCE only needs 1 step. Regarding reagent toxicity, the reagents used for sodium VC-2-phosphate (such as POCl₃ and pyridine) have high toxicity, whereas the reagent toxicity of TVCE is extremely low. For by-products, sodium VC-2-phosphate generates more by-products like phosphate and pyrophosphate, while TVCE produces only one type of by-product (acetic acid). Especially, the acetic acid produced by the reaction can be recovered through distillation, thus achieving the co-production of TVCE and acetic acid, significantly enhancing the atomic economy of the process and eliminating resource waste from the source. In terms of purification methods, sodium VC-2-phosphate needs to adopt column chromatography or ion exchange, while TVCE can complete purification through simple extraction. In terms of Atom Economy [24], sodium VC-2-phosphate is 60%, and TVCE is higher, reaching 83%. In terms of Environmental Factors [25], sodium VC-2-phosphate is 70, and TVCE is only 10. Overall, the synthesis process of TVCE is superior to that of sodium VC-2-phosphate in terms of step simplification, toxicity control, by-product reduction, purification convenience, atomic utilization efficiency and environmental friendliness. In Table 6, the Rf value is a characteristic numerical value used in thin-layer chromatography to describe the degree to which a compound moves on the stationary phase. The magnitude of the Rf value reflects the polarity of the compound, and its value is inversely proportional to the polarity of the compound [26]. Using ethyl acetate:petroleum ether = 1:2 as the developing agent, the Rf values of sodium VC-2-phosphate and TVCE are 0 and 0.69, respectively. This result indicates that the polarity of sodium VC phosphate is much greater than that of TVCE. Sodium VC-2-phosphate is a water-soluble compound with hygroscopicity and strong polarity. TVCE may possess the physiological activities of both VC and acetate, while sodium VC-2-phosphate only has the physiological activity of VC. Although water solubility and strong polarity increase the dissolution rate, they can easily lead to low bioavailability [27, 28]. Moreover, the hygroscopicity can cause caking problems, thereby affecting the uniformity and processing stability of the feed. However, the high lipid solubility and low polarity of TVCE enable it to complete transmembrane transport more effectively through passive diffusion and other means, which is expected to significantly promote its absorption and thereby increase its bioavailability [29, 30]. It is worth noting that the content of VC in TVCE is much higher than those of sodium VC-2-phosphate. In a word, TVCE has more advantages than VC-2-phosphate esters in both synthesis methods and product features.

**Table 4:** Comparison of synthesis methods and technological characteristics of VC-2-phosphate esters and TVCE

Product names	Synthetic methods	Technological characteristics
VC-2-phosphate esters	Phosphorus oxychloride phosphate esterification	Complex operations, involving a variety of organic solvents, many by-products, purification difficulty, environmental pollution, high cost, etc.
VC-2-phosphate esters	Phosphate phosphorylation	Low product content, unstable curing, dark-colored products and poor product uniformity
VC-2-phosphate esters	Phosphorus pentoxide phosphate esterification	Difficult operation, pre-treatment is required before drying, spray drying is required, low efficiency, environmental pollution, and difficulty in large-scale production
VC-2-phosphate esters	VC phosphorylase	Difficult strain induction, long time-consuming, and the method is in the experimental stage and has not yet been industrialized
TVCE	Acetic anhydride esterification	Short route, simple operation, no toxic reagents, less waste liquid, high yield, no purification required, high-purity products, and easy to scale up production

**Table 5:** Comparison of synthesis processes of TVCE and sodium VC-2-phosphate ester

Items	Sodium VC-2-phosphate	TVCE
Reaction steps	2–4	1
Reagent toxicity	High (such as POCl₃, pyridine)	Extremely low
By-products	More (phosphate, pyrophosphate, etc.)	Less (only acetic acid)
Purification methods	Column chromatography/ion exchange	Simple extraction
Atom economy	60%	83%
Environmental factors	70	10

Atom economy = molecular weight of the target product/sum of molecular weights of all reactants × 100%; Environmental factors = total waste (kg)/total product (kg)

**Table 6:** Comparison of characteristics of sodium VC-2-phosphate and TVCE

Items	Sodium VC-2-phosphate	TVCE
Solubility	Water solubility	Lipophilicity
Hygroscopicity	Low	No
Retardation factor (Rf)	0	0.69
Possible bioactivity spectrum	VC	VC and acetate
VC content, %	30–42	50

### Stability assessment of TVCE

The stability of TVCE is evaluated by its performance in artificial SIF and its tolerance to high temperatures. During in vitro simulated digestion, the concentration of TVCE in the digestive fluid is inversely proportional to the digestion time and the amount of pancreatic lipase added. This phenomenon first indicates that TVCE can be hydrolyzed by pancreatic lipase. As a key hydrolase in the intestine, pancreatic lipase is mainly responsible for breaking ester bonds [31]. Since TVCE molecules contain four acetyl groups (ester bonds), they can be recognized and hydrolyzed by pancreatic lipase, thereby releasing free VC and acetate. This inverse proportional relationship also directly proves that pancreatic lipase promotes the hydrolysis of this ester. Secondly, the phenomenon reflects that the hydrolysis rate depends on enzyme concentration and time: it not only conforms to the rule that "the longer the time, the more complete the hydrolysis reaction and the less residual substrate (TVCE)", but also follows the principle of enzyme kinetics (an increase in the amount of enzyme accelerates the hydrolysis rate, leading to more substrate consumption within the same time), which is consistent with the characteristic of enzyme-catalyzed reaction kinetics (e.g., Michaelis–Menten equation) that "the substrate consumption rate is positively correlated with enzyme concentration and reaction time" [32]. At the same time, this also suggests that TVCE may be an excellent prodrug or a protected form. Since VC itself is unstable and prone to oxidation, acetylation can improve its stability (such as enhancing lipid solubility and increasing storage resistance). Moreover, in the digestive system, it can be hydrolyzed by pancreatic lipase in the intestine into bioavailable VC, which indicates that the design of using it as a prodrug (converted into an active form in the body) is reasonable. Acetylation can also protect VC from instability in the upper digestive tract (e.g., in the gastric acid environment) and ensure its effective release in the intestine (under the action of pancreatic lipase). In addition, this phenomenon also provides clues for its absorption mechanism in the intestine: free VC (water-soluble) can be absorbed through specific transporters (e.g., sodium-dependent VC transporter 1/2, SVCT1/2) [33], while its ester form (lipid-soluble) may be absorbed through passive diffusion. The VC released after hydrolysis is more easily absorbed through active transport, which helps to improve bioavailability. Meanwhile, it verifies that the intestine is the main site for its hydrolysis and absorption, and this process depends on pancreatic lipase. In summary, this phenomenon shows that TVCE is an excellent prodrug form, which can be effectively hydrolyzed by pancreatic lipase in the intestine to release active VC, and the hydrolysis process is regulated by enzyme concentration and reaction time, providing a digestive kinetic basis for its application in nutritional supplements or pharmaceuticals.

According to the results of the NMR spectra, TVCE is very stable at 100 °C and 130 °C, and only 6% has decomposed at 160 °C. The results show that TVCE can withstand high-temperature treatment up to 160 °C. The characteristic of TVCE that it can withstand a maximum high temperature of 160 °C has brought a revolutionary breakthrough to the feed industry, fundamentally solving the core problems of ordinary VC in feed processing and storage. In the pelletizing (usually 70–90 °C, sometimes higher) and extrusion (up to 120–150 °C or even higher) processes of feed production, ordinary VC will oxidize and decompose rapidly when the temperature exceeds 70 °C, with a loss rate as high as 50% or even over 80%. This leads to the VC content in the final feed being far lower than the designed value. However, TVCE can pass through these high-temperature processes without loss, ensuring that the VC content in the processed feed meets the standard accurately and breaking through the bottleneck of feed processing technology. In the storage link, feed goes through months of processes such as packaging, transportation, and warehousing from production to being eaten by animals. Ordinary VC is prone to accelerated oxidation and inactivation under the influence of trace elements, minerals, moisture, etc. [34]. Relying on its stable acetylated structure, TVCE is not only heat-resistant but also greatly improves its antioxidant capacity. Its degradation rate is much slower than that of ordinary VC, which significantly extends the product shelf life. This allows distributors and breeders to not worry about the attenuation of VC over time, ensuring that terminal farms obtain effective VC benefits and avoiding potential economic losses. This high-temperature resistance also indirectly ensures the effective dose of VC ingested by animals. Although ordinary VC is added in sufficient quantities in the formula, its actual content may be seriously insufficient after processing and storage, failing to provide animals with the expected health benefits such as anti-stress and immune enhancement. In contrast, TVCE can ensure stability throughout the entire chain from "formula design" to "animal ingestion". It can be enzymatically hydrolyzed to release VC in the intestinal tract, with high bioavailability, and stably exerts physiological functions. In summary, the high-temperature resistance of TVCE is not a simple advantage but a key breakthrough. It solves the long-standing contradiction between "addition" and "actual effect" in the feed industry, realizing zero loss during processing, high stability during storage, and guaranteed ingestion dose. It greatly improves the reliability and consistency of feed products and provides important nutritional support for the intensive and standardized production of the aquaculture industry.

### The growth performance and antioxidant capacity of zebrafish

The VC0.1% group contains 0.35 g/kg of VC, while the TVCE0.036% group simultaneously contains 0.18 g/kg of VC and 0.18 g/kg of acetate. Moreover, the FCR of these two groups shows a similar decline, revealing that TVCE efficacy does not rely entirely on the activity of VC. As mentioned above, after enzymatic hydrolysis in the body, it not only releases 0.18 g/kg of VC but also an equivalent dose of acetate. As a crucial energy source and metabolic regulator for fish, this SCFA can be directly absorbed by the intestines and enter the tricarboxylic acid cycle to provide high-efficiency energy [35]. This reduces the energy consumption of nutrients such as protein, thereby lowering the FCR. Furthermore, there may be a synergistic effect between VC and acetate: the former improves the antioxidant status and metabolic environment, while the latter provides easily utilizable energy substrates. Together, they enhance feed conversion efficiency. This research also challenges the traditional notion that VC sources are evaluated solely based on "VC equivalents". For instance, the theoretical VC supply of the VC0.1% (0.35 g VC/kg) is nearly twice that of the TVCE0.036% group (0.18 g VC/kg), yet their effects are comparable. This indicates that TVCE has a significantly higher bioavailability. Additionally, the greater lipophilicity of TVCE enables it to more easily pass through the lipid bilayer of cell membranes for intestinal absorption, resulting in superior absorption pathways and efficiency compared to the water-soluble sodium VC-2-phosphate [36]. These characteristics also offer key implications for scientific research and production: TVCE should not be regarded merely as a VC source, but more importantly as a "VC-acetate composite precursor". It provides feed formulators with a new idea of using one additive to address multiple demands such as vitamin supplementation and energy optimization. Overall, the efficacy of TVCE stems from the dual contributions and synergistic effect of VC and acetate.

The experiment comprehensively confirmed the excellent antioxidant effect of TVCE from two key organs (intestine and liver) using three classic indicators: SOD, T-AOC, and MDA. This indicates that TVCE can be effectively absorbed and utilized by zebrafish, significantly enhancing the body's intrinsic antioxidant defense system and protecting cell membranes from oxidative damage. The most crucial finding is its higher "bioefficacy", which directly compares the efficiency of two VC sources. Under equivalent VC dosage conditions, the TVCE0.052% group (providing 0.26 g VC/kg) exhibited similar intestinal antioxidant effects (significant increases in SOD and T-AOC, and a significant decrease in MDA) to the VC0.1% group (0.35 g VC/kg). More notably, in the liver, the T-AOC content of the low-dose in TVCE0.036% group (providing only approximately 0.18 g VC/kg) was significantly higher than that in the VC0.1% group (providing 0.35 g VC/kg), demonstrating its higher bioavailability. Mechanistically, while releasing active VC through enzymatic hydrolysis in the body, TVCE also releases acetate in equimolar amounts. As a SCFA, acetate serves as an important energy source for fish and a key metabolic regulatory signaling molecule. It can provide efficient energy for intestinal and liver cells (via the production of acetyl-CoA to enter the tricarboxylic acid cycle), regulate transcription factors (such as the Nrf2 signaling pathway) to upregulate the gene expression of various antioxidant enzymes, and help maintain the integrity of cell membrane structures [37]. This creates a "1 + 1 > 2" synergistic effect with VC, whereas sodium VC-2-phosphate can only provide the single function of VC. This result holds significant guiding value for research and production: it suggests that when selecting VC additives, attention should be paid not only to the "VC equivalent" on the label but also to bioefficacy and comprehensive physiological effects; it enables a substantial reduction in additive dosage (from 0.35 g VC/kg to 0.18–0.25 g VC/kg) to achieve equivalent or even better antioxidant health effects, thereby lowering formula costs; it contributes to improving fish health (enhancing stress resistance) and meat quality (extending the shelf life of aquatic products by reducing lipid peroxidation); and it points out a research direction focused on the synergistic effects of nutrients. In summary, TVCE is a highly effective VC source with proven efficacy, outstanding efficiency, innovative mechanisms, and great value. It provides an optimized solution for aquatic feed formulations and plays a crucial role in promoting fish health and reducing aquaculture costs.

### The effects of different levels of TVCE on tilapia growth, liver and intestinal health

Experiment 1 has confirmed that TVCE is a superior VC source to sodium VC-2-phosphate. The VC and acetate in TVCE can work synergically to improve animal health. In Exp. 2, a preliminary study on the effect and mechanism of acetate in TVCE relative to SA was conducted. The experimental results show that at the appropriate addition amounts of 0.1% and 0.2%, the biological titer and health benefits (promoting growth and increasing SR of tilapia) of TVCE are superior to those of the traditional sodium VC-2-phosphate and the physical mixture of sodium VC-2-phosphate and SA. Besides, WGR in the TVCE0.1% group (providing 1.05 g VC/kg and 0.5 g acetate/kg) is much higher than that in the VC0.3% + SA group (providing 1.05 g VC/kg and 1.05 g acetate/kg), indicating TVCE has a significant dose-saving effect compared with the mixture. The reason why TVCE performs exceptionally well may lie in the fact that its molecular structure is more stable and less prone to degradation during processing and storage. Meanwhile, as a lipophilic derivative, TVCE may be more efficiently absorbed by the intestinal tract through passive diffusion and other pathways, overcoming the absorption limitations of water-soluble SA. In addition, the process of TVCE releasing acetate through enzymatic interpretation in the body is gentler and longer-lasting, which can maintain a more stable blood acetate level and avoid the "roller coaster" effect of rapid absorption and excretion of water-soluble SA, thereby continuously meeting the metabolic needs of the body. The control results of the VC0.3% + SA group also reveal to formulators that they need to focus on the bioefficacy of SA rather than simply conducting equivalent substitution. In future, the experiment has verified the effectiveness of SCFAs esterification technology in improving stability and utilization rate, providing a direction for the research and development of other SCFAs related technologies. Therefore, this result shows that the upgrade of SA in aquatic feed from traditional salts to TVCE is a "cost-reducing and efficiency-enhancing" technological innovation. It can simultaneously improve the WGR and SR of tilapia with a lower dosage, driving the application of SCFAs to shift from pursuing the added amount to pursuing "effective supply".

Further, clear research findings on tilapia liver, intestine and gut microbiota indicate that, under the premise of providing an equivalent amount of VC, TVCE (especially at the 0.1% addition level) is more effective than both the mixture of sodium VC-2-phosphate and sodium acetate, and sodium VC-2-phosphate alone, in improving the liver function of tilapia, enhancing the integrity of the intestinal physical barrier, and significantly optimizing the structure of the intestinal flora. This lays a solid physiological foundation for enhancing their growth performance and SR [15, 38]. Specifically, the serum ALT level and liver histology score in the TVCE0.1% group exhibit downward trends, and the hepatic TG level in TVCE groups are lower than that in the VC0.3% + SA group. This directly proves that TVCE greatly alleviates liver burden or damage, resulting in more intact liver cell membranes and better liver function. The reason lies in the fact that the traditional mixture of sodium VC-2-phosphate and sodium acetate, or sodium VC-2-phosphate alone, may have low bioavailability in the body or produce additional metabolic burden (with the mixture showing a more pronounced effect). In contrast, relying on its stable properties and efficient absorption and metabolism pathway, TVCE provides the liver with a "highly efficient and mild" supply of acetate. Meanwhile, a significant reduction in serum DAO levels was observed in the TVCE0.1% and TVCE0.2% groups relative to the VC0.3% + SA group. When combined with the results of intestinal section analysis and histology score, it indicates the beneficial effect of TVCE on intestinal health [39]. Additionally, the scores of liver and intestinal sections [40] in TVCE0.1% group were reduced compared with those in the VC0.3% + SA group, which, from the perspective of histomorphology, suggests that hepatocytes were in a better state, and intestinal villi were denser with a larger absorption area. This forms a complete "biochemical index-histomorphology" evidence chain. The optimization of intestinal flora structure is the foundation of health benefits. An increased ratio of (acid-producing bacteria phyla/Proteobacteria phyla) signifies the transition of the flora from a "pathogenic type" to a "healthy type" [13]. The elevated abundance of Bacteroidetes phylum and the rise in flora health index indicate the proliferation of beneficial acid-producing bacteria and the inhibition of potential pathogenic bacteria. The underlying mechanism is as follows: when TVCE is digested in the intestine or fermented by the flora, it not only provides VC but also releases acetate (the TVCE0.1% group provides 0.5 g/kg of acetate) which acts as a prebiotic. This acetate selectively promotes the proliferation of beneficial acid-producing bacteria. The SCFAs produced in this process can lower the intestinal pH to inhibit pathogenic bacteria, serve as an energy source for intestinal cells to repair the intestinal barrier, and alleviate liver metabolic pressure after being absorbed. 0.1% TVCE is presumably the optimal balance point for the efficient supply of VC and the appropriate supplementation of acetate. The results show TVCE is a "functional" nutrient that integrates vitamin supplementation, intestinal flora regulation, and liver-gut protection. It can proactively promote organ health and enhance body functions, providing data support for the upgrading of SCFAs sources in aquatic feed. The research reveals a new pathway for improving the health of farmed animals by targeting intestinal microecology through nutritional strategies and relying on the "microbiota-gut-liver" axis. It also suggests the TVCE0.1% group (0.5 g acetate/kg) as the optimal addition level. This group outperforms the VC 0.3% + SA group (1.05 g acetate/kg), proving that TVCE is more efficient and that there exists an "optimal acetate stimulation dose" (1 + 1 > 2), which provides a scientific basis for precise cost reduction. Moreover, it clearly reveals the internal mechanism by which TVCE promotes growth: protecting the liver, repairing the intestine and optimizing the structure of the intestinal flora to improve the efficiency of nutrient digestion and absorption as well as metabolic health. In practical production, TVCE can replace the traditional combination of VC sources and organic acids at a lower addition level to reduce feed costs. It can also serve as a daily flora management strategy to reduce reliance on antibiotics and chemical drugs, facilitating green and sustainable aquaculture. Additionally, it provides feed enterprises with an ideal core additive option for developing high-end functional feeds such as "liver-gut protective type" and "stress-resistant type".

To sum up, this study suggests from three dimensions: physiology and biochemistry, histomorphology, and microbial ecology that TVCE is a highly effective additive capable of systematically improving the health of tilapia. TVCE significantly optimizes the acetate utilization efficiency of tilapia on the basis of equivalent VC supply. It achieves outstanding improvement in tilapia liver and intestinal health through the synergistic effect of VC, which is the fundamental reason for its ability to enhance tilapia growth performance and SR. This provides a solid theoretical basis for the wide application of TVCE as an efficient and functional acetate source in aquatic feed.

### The intrinsic connection between synthetic processes, product characteristics and biological effects

The one-step synthesis process adopted in this research is not merely for simplifying the workflow. Its main value lies in the creation of a brand-new molecular entity (TVCE), which enables the synchronous and in-situ release and absorption of VC and acetic acid in animal bodies. In contrast, due to the differences in their physicochemical properties (water solubility vs. fat solubility and absorption rate), physical mixtures tend to go their separate ways during processing and digestion. This unique property of TVCE serves as the material basis for achieving the synergistic effects of VC and acetic acid (such as promoting growth and improving liver and intestinal health), a true synergy that cannot be attained through simple physical mixing. The high lipid solubility and low polarity of TVCE, conferred by its synthetic process, are the keys to its exceptional biological potency. This characteristic enables it to efficiently penetrate the lipid bilayer of intestinal epithelial cells through passive diffusion, overcoming the limitations of water-soluble VC derivatives (such as sodium VC-2-phosphate) [41] and SA [42], which rely on active transport or pore absorption. In this way, TVCE achieves higher absorption efficiency and bioavailability. This directly explains why, in tilapia experiment, TVCE, even at a lower addition level, demonstrates superior effects to traditional additives in promoting growth and protecting liver and intestinal health. Traditional VC and SA suffer from significant loss during feed processing (pelleting or extrusion) and storage. This discrepancy leads to a severe mismatch between the "theoretical added amount" in formulations and the "actual effective amount" ingested by animals. The TVCE synthesized in this study, with its high-temperature stability at 160 °C, ensures zero or extremely low loss of active ingredients throughout the entire chain—from the feed production line to the intestine of tilapia. Therefore, the significant effects observed in the tilapia experiment are based on the real and stable presence of TVCE in the feed, which verifies the great application value of this synthesis process in addressing the pain points of the industry. The neutral TVCE molecules obtained via our synthesis process avoid the potential interference of traditional sodium salt additives on intestinal osmotic pressure and ion balance. A more stable intestinal environment is conducive to the colonization and growth of beneficial microorganisms. This provides a reasonable explanation for the results observed in the experiment, which TVCE optimizes the intestinal flora structure of tilapia (e.g., increased abundance of Bacteroidetes). The process design avoids the potential negative ecological impacts of salt-based additives at the molecular level, thereby being more beneficial to intestinal health.

## Conclusions

In this study, TVCE was synthesized successfully, and its physicochemical properties, stability and biological effects were systematically evaluated. The results show that TVCE has the advantages of simple synthesis process, high yield and good thermal stability, and can effectively overcome the problems of poor stability and low bioavailability existing in the application of traditional VC and SA in aquatic feed. Animal experiments have confirmed that TVCE not only significantly enhances the antioxidant capacity of fish, but also promotes growth, improves liver and intestinal health, and exerts a synergistic nutritional effect by regulating the structure of intestinal flora. Compared with the physical mixture of sodium VC-2-phosphate and SA, TVCE can achieve even better physiological effects at a lower addition amount, demonstrating higher biological titer. Therefore, TVCE with high stability, efficient utilization and synergistic physiological activity was created through a simple and green synthetic process. Its outstanding effect as a substitute for traditional VC and SA was verified through animal experiments, providing a full-chain solution from molecular design to efficacy verification for solving key technical problems in the aquatic feed industry.

## Supplementary Information

Additional file 1. Table S1. The effects of TVCE on SR, WGR and FCR of zebrafish fed experimental diets for 2 weeks.

## Data Availability

No datasets were generated or analysed during the current study.

## Abbreviations

ALT: Alanine aminotransferase
DAO: Diamine oxidase
FCR: Feed conversion ratio
FT-IR: Infrared spectroscopies
HPLC: High performance liquid chromatography
MDA: Malondialdehyde
MS-222: Tricaine methanesulfonate
NMR: Nuclear magnetic resonance
Rf: Retardation factor
SA: Sodium acetate
SCFAs: Short-chain fatty acids
SGR: Specific growth rate
SIF: Simulated intestinal fluid
SOD: Superoxide dismutase
SR: Survival rate
SVCT1/2: Sodium-dependent VC transporter 1/2
T-AOC: Total antioxidant capacity
TG: Total triglycerides
TVCE: Tetraacetyl vitamin C ester
UV-vis: Ultraviolet-visible
VC: Vitamin C
WGR: Weight gain rate
