# Supplementary material for: Synthesis and efficacy of tetraacetyl vitamin C ester: a novel alternative to traditional vitamin C and sodium acetate in aquafeeds
Source: J Anim Sci Biotechnol. 2026 Apr 24;17:77. doi: 10.1186/s40104-026-01394-y (PMC13107756; doi:10.1186/s40104-026-01394-y)
Supplement: Supplementary file 1 — Additional file 1. Table S1. The effects of TVCE on SR, WGR and FCR of zebrafish fed experimental diets for 2 weeks. [file 40104_2026_1394_MOESM1_ESM.docx]

**Table S1** The effects of TVCE on SR, WGR and FCR of zebrafish fed experimental diets for 2 weeks

| **Items** | **Control** | **VC0.1%** | **TVCE0.036%** | **TVCE0.052%** | **TVCE0.106%** |
| --- | --- | --- | --- | --- | --- |
| SR, % | 97.50±5.00 | 100 | 100 | 100 | 100 |
| WGR, % | 67.91±6.09 | 71.16±1.36 | 71.79±1.49 | 70.06±7.29 | 70.14±4.20 |
| FCR | 1.37±0.10 | 1.28±0.03 | 1.27±0.03 | 1.31±0.12 | 1.30±+0.08 |

*SR* Survival rate, *WGR* Weight gain rate, *FCR* Feed conversion ratio

Values are presented as mean ± SEMs (*n* = 4 biological replicates)
